# Predicting recovery after stressors using step count data derived from activity monitors

**DOI:** 10.1038/s41746-025-01998-0

**Published:** 2025-10-09

**Authors:** Dario Baretta, Sarah Koch, Joren Buekers, Judith Garcia-Aymerich, Lenka Knapova, Steriani Elavsky, Job Godino, Merlijn Olthof, Anna Lichtwarck-Aschoff, Ruud den Hartigh, Guillaume Chevance

**Affiliations:** 1 Institute of Psychology, University of Bern, Bern, Switzerland; 2 University of Basel, Basel, Switzerland; 3 ISGlobal, Barcelona, Spain; 4 CIBER Epidemiología y Salud Pública (CIBERESP), Barcelona, Spain; 5 Universitat Pompeu Fabra (UPF), Barcelona, Spain; 6 University of Ostrava, Ostrava, Czechia; 7 Family Health Centers of San Diego, San Diego, CA USA; 8 UC San Diego, San Diego, CA USA; 9 Faculty of Behavioural and Social Sciences, University of Groningen, Groningen, The Netherlands; 10 Univ Rennes, Inserm, EHESP, Irset (Institut de recherche en santé, environnement et travail), Rennes, France

**Keywords:** Psychology, Human behaviour, Lifestyle modification

## Abstract

This study examines the stressor-response process in physical activity among 226 participants across four countries. We analyzed their step count collected via activity monitors before and after a significant stressor: the COVID-19 lockdown. Results showed that a ‘local dynamic complexity’ metric significantly predicts the rate of recovery to pre-COVID levels of physical activity. These findings provide new opportunities for just-in-time interventions to support physical activity recovery after disruptive stressors.

## Introduction

**Fig. 1:**
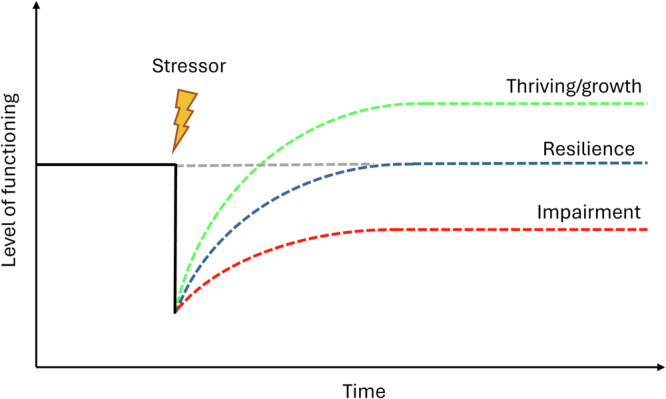
Potential responses to perturbations generated by a stressor. The black solid line represents a person’s state of functioning before encountering a stressor. Figure adapted from ref. ^6^. Copyright 2010 by John Wiley and Sons. Used with permission.

Physical activity is key to health and wellbeing^1^. However, this behavior is sensitive to numerous stressors which can perturb regular engagement. By stressors, we refer to external or internal events that can disrupt a system’s equilibrium, necessitating adaptive responses to restore balance^2,3^. According to ecological models of physical activity^4^, stressors can occur at the intra-individual or extra-individual level. Intra-individual stressors originate from biological, psychological, or behavioral factors (e.g., illness or psychological distress)—whereas extra-individual stressors stem from the external environment, including the social, natural, and built physical settings, as well as policy regulations, all of which may hinder physical activity. Previous research—drawing from both broader resilience frameworks^2,5,6^ and empirical studies^3^—has suggested that individuals’ may respond differently to such perturbations: some may not return to prior physical activity levels (i.e., impairment), others may successfully recover (i.e., resilience), and some may even increase beyond their previous functioning (i.e., thriving, growth) (Fig. 1). Recent advances in mobile sensing have enabled precise characterization and quantification of these stressor-response trajectories through the analysis of high-resolution time series data^2,5^. Among various outcomes, data from activity monitors, particularly step count, have emerged as powerful indicators of an individual’s physical state throughout the stressor-response trajectory (e.g.,^7^).

**Fig. 2:**
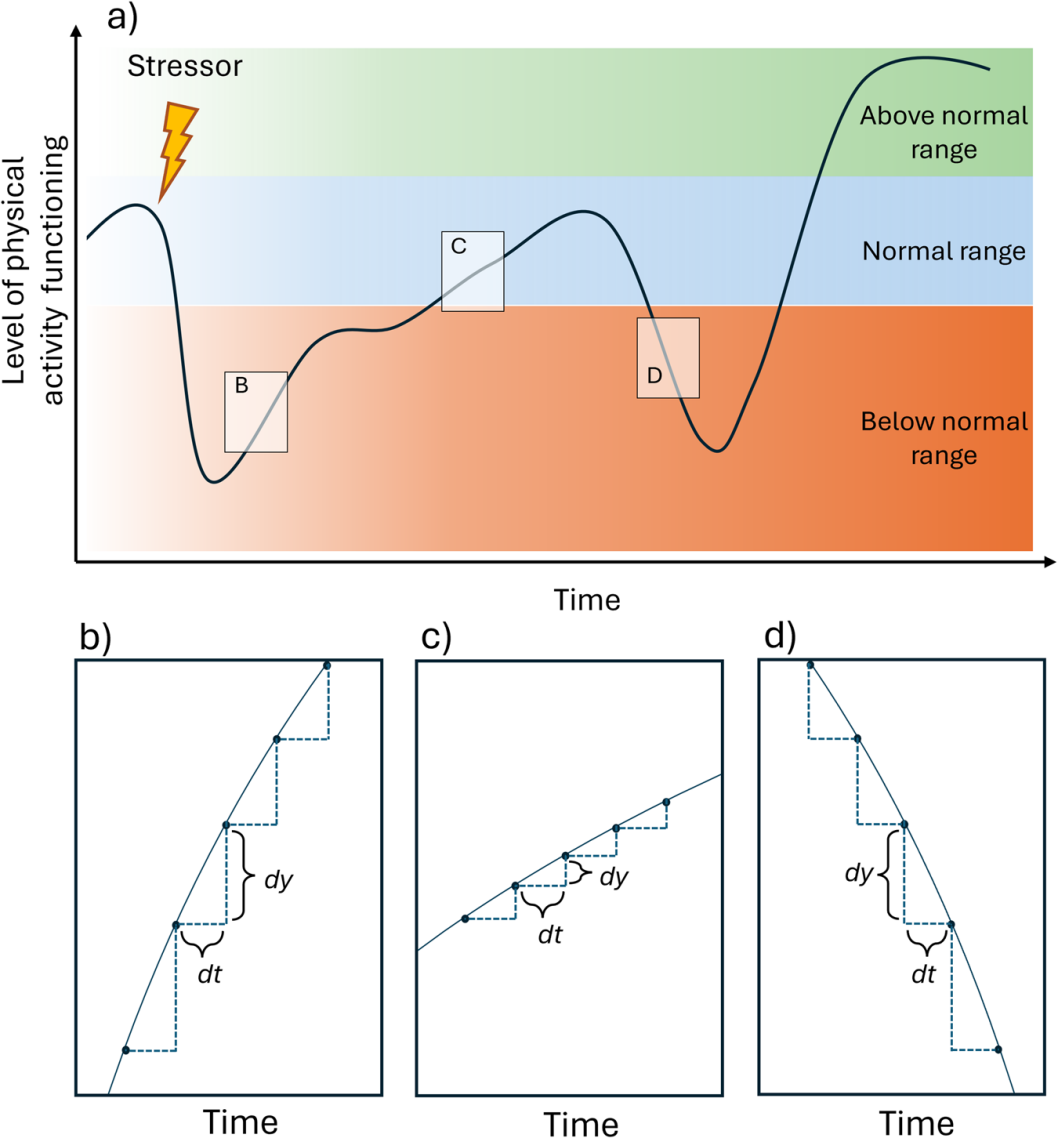
A physical activity stressor-response process characterized by varying direction and pace. **a** Conceptual visualization of various responses to stressors, with the overall response trajectory characterized by varying momentary pace and direction. **b**−**d** The within-person progression throughout the response process can be characterized by different rates of change, indicating whether the momentary pace is fast or slow (**b** vs. **c**), positive or negative (**b, c** vs. **d**). The rate of change, d*y*, reflects how much the outcome variable (here, *step count*) changes in response to a one-unit change, d*t*, in the independent variable (here, *time*). The rate of change can be operationalized as the first-order derivative of a growth function fitting the outcome variable. It can take positive, negative, or zero values depending on whether the change is positive, negative, or null.

Step count is a passive digital biomarker that provides a scalable, unobtrusive, time-sensitive, and cost-effective way to continuously monitor individuals’ physical activity. While it does not capture every dimension or type of physical activity, it represents a direct and pragmatic indicator of daily movement patterns. Specifically, it enables researchers to capture key features of the stressor-response trajectory, such as its immediate direction (e.g., increasing vs. decreasing) and momentary pace (Fig. 2). Direction and pace may vary over time, with reversals or temporary slowdowns in trajectory resulting from interactions with additional stressors that can operate at different levels. Tracking day-to-day changes in step count offers the opportunity to model and quantify the dynamics that govern how individuals respond to stressors. Ultimately, predicting future momentary direction and pace of the stressor-response trajectory (Fig. 2b−d) would enable the development and optimization of just-in-time intervention strategies—timely, targeted support provided at critical moments^8^—that could accelerate recovery and prevent delays or relapses that might occur in the weeks following the stressor.

While ecological models highlight the multilevel influences on physical activity, they offer limited tools for capturing the dynamic and nonlinear patterns of individual responses over time. Complex systems theory complements this perspective by providing a framework to model and predict individual progression throughout their response to stressors^9–11^, particularly through early warning signals. Statistical markers of early warning signals include increases in autocorrelation, variance, and dynamic complexity (DC)^11,12^. In particular, increased DC indicates the system’s heightened sensitivity to external influences (see “Methods” section) and has recently been demonstrated to be a predictor of future losses in step count^13,14^.

Leveraging mobile sensing and grounded in the complex systems framework, this study aims to shed light on the largely unexplored stressor-response process. To achieve this, we conducted a multi-country study focusing on this process by analyzing the step counts of individuals following a significant stressor: the COVID-19 lockdown and its associated restrictions (e.g., enforced home confinement)^15^. Specifically, we investigated whether DC in step count, as measured by activity monitors, could predict the slowdowns (Fig. 2c vs. Fig. 2b) or even reversals (Fig. 2d vs. Fig. 2b–c) in the stressor-response trajectory, also derived from step counts. We hypothesized that increases in local DC would be associated with a decrease in the local rate of change d*y* in daily step counts in the following days.

To test this hypothesis, we analyzed the stressor-response trajectory in daily step count following the first COVID-19 lockdown across 226 participants (77 from the United States, 65 from the Czech Republic, 48 from Spain, and 36 from Norway), resulting in a total of 44,825 daily step count observations from activity monitors (e.g., Fitbit, Garmin). Before the lockdown, the median daily step count was 10,167 (IQR: 8063–12,188; mean = 10,544), suggesting that the current sample was generally more active than the broader adult population and fell just below the highest quartile of daily step count reported in previous meta-analytic research^16^. During the 30 days following the lockdown, the median perturbation in daily steps was 3744 (IQR: 2312–5252; mean = 4063). Consequently, on average, participants shifted from taking over 10,000 steps per day to approximately 6500 steps per day, which falls below the recommended target for health benefits (e.g., 9000 steps per day,^16,17^). For 110 participants (49%), it took a median of 88 days (IQR: 50–130; mean = 93) to return to pre-lockdown levels of daily steps, while 116 participants (51%) had not returned by September 30, 2020 (ca. 6.5 months after the first lockdown was issued). The average median rate of change in daily steps following the stressor was 11 steps per day, indicating that, in median, participants regained 11 steps per day as part of their response to the stressor (see Supplementary Table 1 for descriptive statistics of key variables by data source).

**Fig. 3:**
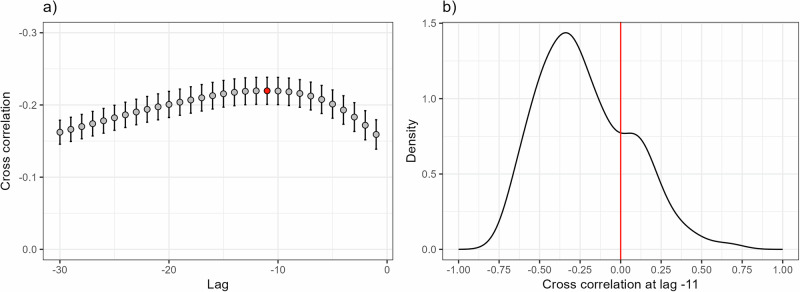
Cross-correlation between local DC and rate of change. **a** Shows the cross-correlation values between local DC and rate of change at various lags. **b** Shows the sample distribution of the cross-correlation values at lag-11.

After computing individuals’ daily rate of change in step count, we computed a local DC score to characterize critical fluctuations in the step count time series^18^. Local DC (independent variable) was used to predict the rate of change in the stressor-response trajectory (dependent variable). Then, we explored the cross-correlation between local DC and the rate of change in daily step count to identify the lag with the highest correlation. The average cross-correlation was checked from a 1-day lag to a 30-day lag. Results, shown in Fig. 3, indicate that the highest average correlation occurred at lag-11 (*r* = −0.22).

**Fig. 4:**
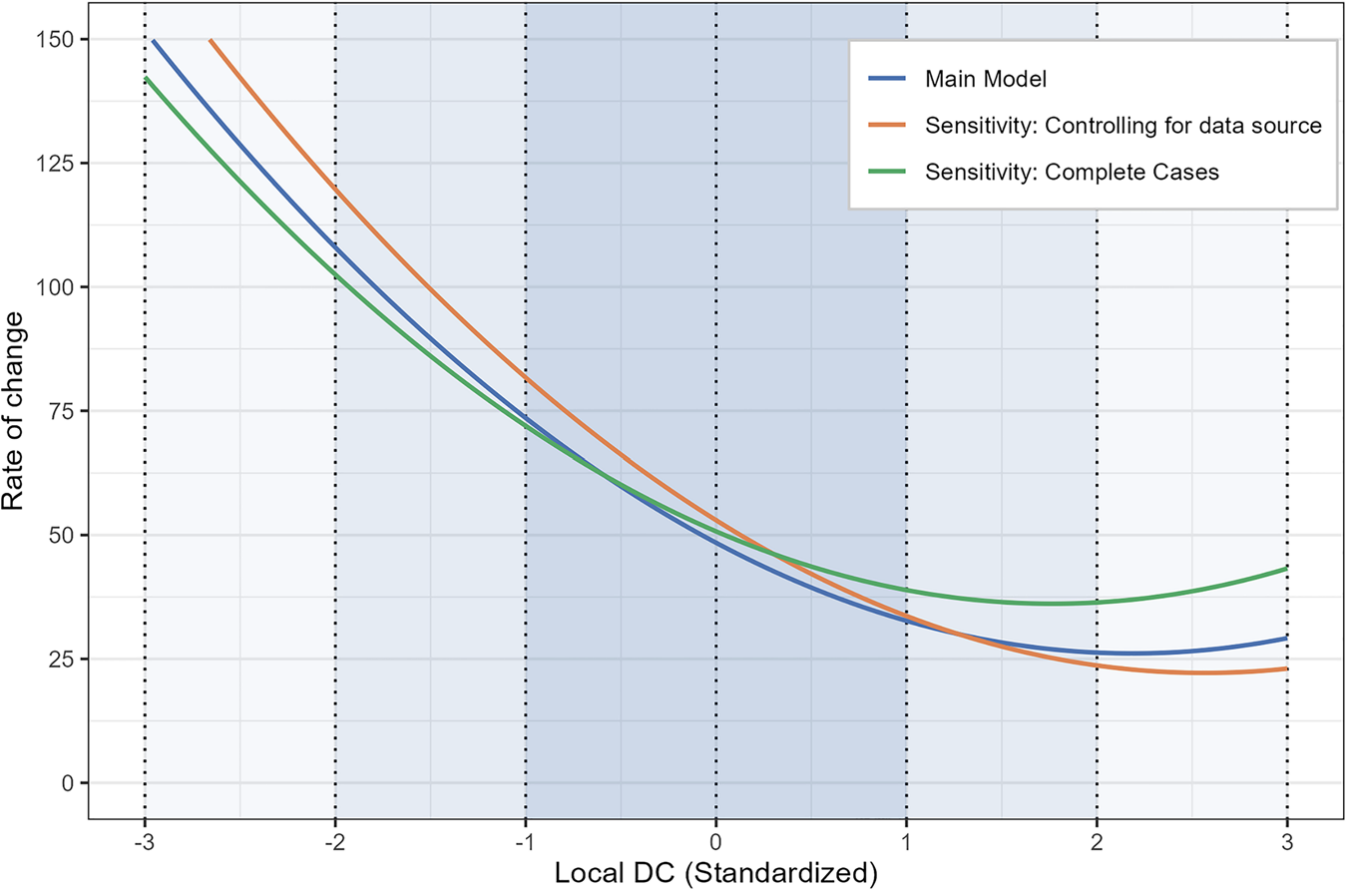
Non-linear association between local DC and the rate of change in daily step count. The three lines represent the modeled non-linear association (combining linear and quadratic terms) between local DC and the rate of change in daily step count. The blue line corresponds to the main model (Table 1), the orange line to the model controlling for the moderating effect of the United States-based Smart 2.0 study (Supplementary Table 2), and the green line to the complete case analysis (Supplementary Table 4). The rate of change values at local DC = 0 reflect each model’s specific intercept. The associations between local DC and rate of change are interpreted with the time effect held constant at zero.

After identifying the time lag at which local DC had its largest association with the rate of change, we conducted multi-level analyses to quantify the association between local DC at lag-11 and future rate of change in step count. The multilevel model included a random intercept for each participant and random slopes for three continuous independent variables: time, local DC, and the quadratic term of local DC. We found a negative effect of time (*B* = −43.51, 95% CI [−53.98, −33.03]), indicating that the rate of change decreased as more time passed since the stressor occurred (see Table 1). Local DC was a significant predictor of the rate of change, with a negative linear effect (*B* = −20.42, 95% CI [−25.78, −15.07]) and a positive quadratic effect (*B* = 4.67, 95% CI [2.44, 6.90]), suggesting that the negative association weakens at higher levels of local DC. Based on these estimates, the overall effect of local DC is -15.75 at one standard deviation above the mean, and 25.09 at one standard deviation below the mean (see Fig. 4 and Supplementary Fig. 1). To help contextualize the clinical relevance of these findings, it is useful to consider the distribution of local DC values across the observation window. Participants had, on average, 28 days with local DC values exceeding one standard deviation above the mean, which together predicted a cumulative delay of about 440 daily steps (28 days × −15.75 steps) in the recovery trajectory by the end of the study period. Conversely, they had an average of 24 days with local DC values falling one standard deviation below the mean, which together predicted a cumulative acceleration of about 600 daily steps (24 days × 25.09 steps).

**Table 1:** Multilevel model summary

	Fixed effects		Random effects
DV is rate of change	Est. (SE)	95% CI	SD
**Intercept**	**48.45 (4.89)**	**[38.85, 58.04]**	70.89
**Time**	**−43.51 (5.35)**	**[−53.98, −33.03]**	78.42
**Local DC**	**−20.42 (2.73)**	**[−25.78, −15.07]**	39.85
**Local DC^2**	**4.67 (1.14)**	**[2.44, 6.90]**	16.11
**Pre-lockdown median steps**	−0.81(1.19)	[−3.15, 1.52]	**–**
Number observations = 39,401; ICC = 0.3; R2 Marg. = 0.067; R2 Cond. = 0.325			

Values presented in bold are significant based on their 95% confidence intervals; *R*^2^ Marginal = the part of the variance in the outcome explained by the fixed effects; *R*^2^ Conditional = the part of the variance in the outcome explained by the full model.

Sensitivity analyses were conducted to evaluate the robustness of the current findings (Fig. 4 and Supplementary Tables 2–6). We tested whether the association between local DC on the rate of change was moderated by the data source (see Table 2 for an overview of included datasets). The results indicated that this association was stronger when controlling for the moderating effect of the United States-based Smart 2.0 study (i.e., the association was weaker in this data source). Specifically, the linear effect was *B* = −24.01 (95% CI [−29.97, −18.05]) and the quadratic effect was *B* = 4.68 (95% CI [2.45, 6.91]), resulting in a combined effect of −19.33 at one standard deviation above the mean and 28.69 at one standard deviation below the mean. An additional sensitivity analysis based solely on complete cases (i.e., excluding missing values) showed a reduced but still significant effect (combined estimate = −13.03 at one standard deviation above the mean and 20.00 at one standard deviation below the mean).

**Table 2:** Data sources information

	Study			
	SMART 2.0^26^	4HAIE^22^	COVICAT^24^	Open dataset^23^
**Study characteristics**	A 24-month (96 weeks), three-arm, parallel-group randomized controlled trial targeting weight loss among young adults with overweight or obesity.	A 12-month prospective cohort study, examining physical activity in an air-polluted environment.	A cohort study to characterize the health impact of the COVID-19 pandemic on the population. These data were retrospectively collected using crowdsourcing from participants of the cohort GCAT cohort^45^.	An open dataset providing physical activity data during the COVID-19 pandemic. Two years of data were automatically downloaded from consenting participants by directly accessing the Garmin and Fitbit cloud storages.
**Data collection**	Between April 2019 and February 2024, in San Diego, USA. Data collection was prospective.	Between April 2019 and September 2022, in the Moravian-Silesian and South Bohemian Regions of Czech Republic. Data collection was prospective.	Between September 2019 and October 2020, in Catalonia (Spain). Data collection was retrospective.	Between January 2019 and December 2020, in Norway. Most of data collection was retrospective.
**Ethical approval**	Study approved by the Human Research Protections Program at UC San Diego (protocol #181862).	Study approved by the Ethics Committee of the University of Ostrava, Czech Republic.	Study approved by the Parc de Salut Mar Ethics Committee (CEIM-PS MAR, no. 2020/9307/I) and the Hospital Universitari Germans Trias i Pujol Ethics Committee (CEI no. PI20-182)	Study reviewed by The Regional Committees for Medical and Health Research Ethics North (reference 164780) and the Norwegian Centre for Research Data (reference 628485)
**Date of the first COVID-19 lockdown**	March 19th, 2020	March 16th, 2020	March 15th, 2020	March 12th, 2020
**Original sample size (included in this study)**	638 (77)	1314 (65)	240 (48)	113 (36)
**Activity monitor**	Fitbit Charge 3	Fitbit Charge 3 and 4	Fitbit, Garmin, Withings, GoogleFit, Nokia, or Oura	Fitbit (35%) Garmin (65%)
**Demographics of the participants included in the study**^a^	Biological sex: 45 (58%) Female Age: 24 (5)	Gender: 33 (51%) Female Age: 39 (9)	Gender: 24 (50%) Female Age: 53 (7)	Gender: 59 (56%) Female Age: 41 (11)
**Wear time information**	Available	Available	Not available	Available only for Fitbit users
**Definition of valid days for step count data**	At least 600 minutes of valid minute heart rate signal per day	At least 600 minutes of valid minute heart rate signal per day	At least one recorded step count.^b^	Fitbit users: At least 600 minutes of valid minute heart rate signal per day Garmin users: At least one recorded step count.

^a^N (%); Mean (SD). For the open dataset, individual participant characteristics could not be linked to their respective physical activity data. Therefore, summary statistics are reported for the whole sample.

^b^We did not apply a higher step count threshold (e.g., 150 daily steps) because, in cases where both daily step counts and wear time data were available (e.g., Smart 2.0 and 4HAIE studies), there were instances where wear time exceeded 10 hours, but daily steps ranged between 1–150. To account for such scenarios, we set the threshold to zero. This approach accounts for potential days spent in bed, such as during illness (e.g., COVID-19).

In summary, this study examines how step count data from activity monitors can characterize and predict the stressor-response trajectory in physical activity. Building on previous work^13,14^, we found that local DC significantly predicted the rate of change in daily step count after a significant stressor (i.e., the COVID-19 lockdown). This association was non-linear, with lower local DC values being a stronger predictor of accelerated recovery than higher local DC values were of slowdowns (see Fig. 4). These effects are clinically meaningful, particularly when compared to the 3500 steps needed, on average, to return to pre-stressor levels in our sample or the roughly 2500 steps required to meet the recommended target of 9000 daily steps^16,17^. Both elevated and reduced local DC may therefore function as early warning signals, respectively indicating increased risk of slowdowns or greater potential for recovery in the stressor-response trajectory in the following days.

When interpreting these findings, it is important to acknowledge some limitations. First, while aggregating data from multiple studies is a key strength of this work, it also introduces potential biases due to heterogeneity in study-specific characteristics, such as study design, data collection protocols, and country-specific lockdown measures. Although we conducted a moderation analysis to address this issue, fully disentangling the factors underlying differences across studies (e.g., geographic location versus study design) was not possible. Moreover, harmonizing demographic characteristics that may have influenced the rate of change—such as socioeconomic status—was not feasible across studies. Second, the lack of wear time data in some datasets limited our ability to accurately identify and exclude invalid step count observations. Additionally, although the activity trackers used to assess step count (e.g., Fitbit, Garmin) have the advantage of being widely accessible and generally provide accurate step count metrics, they do not match the precision of gold-standard research tools^19–21^, and their proprietary algorithms limit transparency and replicability. Finally, the relatively active nature of the sample may constrain the generalizability of the findings to less active populations. This characteristic likely stems from study-specific inclusion criteria (e.g., a portion of the sample recruited from a runner population)^22^, as well as from the fact that other participants were already using activity trackers^23,24^, which is often associated with a greater propensity to pursue specific goals, such as improving health^25^.

Altogether, the findings of this study underscore the potential of local DC to predict progression throughout the physical activity response to stressors. This metric fits particularly well in contexts where the individual response to major stressors (e.g., the lockdown) is shaped by interactions with additional influences at different levels (e.g., the work or family environment) or with intra-individual factors (e.g., reduced psychological resources), as suggested by ecological models of physical activity^4^. Importantly, local DC is derived entirely from sensor-based data and does not require additional participant resources, such as frequent questionnaires, which are often a barrier in long-term interventions and health surveillance. This early warning signal thus offers valuable opportunities for developing just-in-time interventions that provide timely support during critical stages in the recovery process^8^. For example, when local DC shows an increasing trend indicating instability, an intervention could be triggered to support planning of structured activity routines or initiate goal-setting strategies. However, future empirical work is warranted to evaluate whether just-in-time intervention strategies triggered by changes in local DC can improve recovery after stressors.

Replicating this approach in additional contexts where a successful response to stressors is crucial (e.g., after surgery, during cardiac rehabilitation, following chemotherapy) is necessary to validate these findings across diverse settings. Moreover, extending this approach to other physical activity signals (e.g., minutes of moderate-to-vigorous physical activity) or integrating additional passive data—such as sleep, heart rate variability, GPS, or weather—with local DC derived from step count could help develop more specific early warning signals and enhance our ability to understand and predict stressor-response trajectories. For example, a concurrent rise in DC and physiological stress indicators (e.g., reduced heart rate variability) or disrupted sleep might signal a different type or severity of vulnerability than DC changes observed in isolation. Alternatively, these additional data streams could be combined to derive a multidimensional DC score, rather than calculating it solely based on step count. The increasing availability of such multimodal data presents a promising opportunity to develop more nuanced models of stressor response that integrate behavioral, physiological, and environmental dynamics—ultimately informing more tailored and effective intervention strategies.

## Methods

### Data sources

This study combined physical activity time series collected in the period between October 1st, 2019 and September 30th, 2020 as part of various studies as summarized in Table 2. Each study received ethical approval and written, informed consent to participate was obtained from all participants^22–24,26^.

### Location-specific COVID-19 context

#### Smart2.0 (San Diego, California, US)

In response to COVID-19, a mandatory statewide stay-at-home order was implemented on March 19, 2020, resulting in the closure of gyms, beaches, parks, and recreational spaces. However, beaches and parks were reopened on April 27, 2020^27^. In early May 2020, California launched a reopening program, with implementation delegated to individual counties. In late August, the state introduced a risk-based tier system to guide the tightening or easing of COVID-19 prevention measures in each county.

#### 4HAIE (Czech Republic)

The lockdown in Czechia began on March 16, 2020, with the implementation of restrictions on non-essential travel and the closure of borders with neighboring countries. Travel for work, family, healthcare, and necessities was permitted, as was outdoor physical activity, provided social distancing was observed. Restaurants, cafes, gyms, and most shops were closed. The lockdown officially ended on May 5, 2020, with restrictions being gradually lifted throughout the summer. However, starting in September, some restrictions, such as social distancing and masking, were reinstated incrementally^28^.

#### COVICAT (Catalunya, Spain)

Catalunya entered its first COVID-19 state of emergency on March 14, 2020, enforcing strict home confinement until May 7, 2020. This was followed by a deconfinement period until June 21, 2020, during which various mobility restrictions, such as night curfews, city- or province-wide confinement, physical distancing, mask mandates, and leisure center closures, were imposed. A second state of emergency was declared on October 15, 2020.

#### Open dataset (Norway)

The COVID-19 lockdown in Norway began on March 12, 2020, with the closure of gyms, schools, universities, kindergartens, and similar facilities. Individuals were encouraged to minimize group interactions, although outdoor exercise remained permitted. Social restrictions were progressively eased during April and May but were reintroduced incrementally starting in August 2020^23^.

### Participants

#### Smart 2.0

Participants (*N* = 638) were young adults (aged 18–35 years) recruited from three universities and five community colleges in San Diego, USA. The inclusion criteria included, (1) overweight or obese (25 ≤ BMI < 40 kg/m^2^); (2) available for a 24-month intervention, (3) affiliated with one of the target universities as a student, staff, or alumni, (4) willing and able to use social media, a smartphone, text messaging, and Fitbit devices and app, and (5) willing and able to engage in moderate to vigorous physical activity. The exclusion criteria included, (1) comorbidities of obesity or conditions that prohibit compliance with the study protocol, (2) a recent cardiovascular event, (3) currently being treated for malignancy and/or an eating disorder, (4) planning to have weight loss surgery or enroll in a weight loss program, (5) loss of more than 15 pounds within the past 3 months, and 6) pregnancy or planning pregnancy within 24 months.

#### 4HAIE

A total of *N* = 1314 participants aged 18–65 years were recruited for this study. Of these, *n* = 747 (57%) were physically active runners and *n* = 567 inactive controls (established at baseline by self-report). The participants were split between two regions of the Czech Republic. A highly polluted industrial region (Moravian-Silesian Region): *n* = 750 (*n* = 436 runners, *n* = 314 inactives) and a low-pollution level region (South Bohemian Region): *n* = 564 (*n* = 311 runners, *n* = 253 inactives). Inclusion criteria included (i) age between 18 and 65, (ii) permanent residency in either Moravia Silesia or Southern Bohemia regions for the past 5 years and no plan to move within a year, (iii) owning a smartphone and having internet access, (iv) being a non-smoker. The runners were defined as (i) regularly running for more than 6 weeks with at least 10 kilometers per week, (ii) fulfilling the weekly WHO PA recommendations^29^, (iii) planning to continue running for the next 12 months. The inactive group included individuals who were capable of normal PA including running (i.e., no physician-diagnosed restrictions), but not meeting the recommended levels of PA.

#### COVICAT

The COVICAT study (N = 10,087) is a population-based investigation aimed at assessing the health impact of the COVID-19 pandemic on adults in Catalonia, Spain. It was built upon five pre-existing cohort studies established before the outbreak and inclusion criteria differed between each of the included cohorts. A description of each cohort including their inclusion criteria can be found here^30^. All COVICAT participants were asked to fill out questionnaires and provided blood samples between May and December 2020 as part of the first assessment. Of the 10,087 individuals participating in the first wave of COVICAT, 2801 gave consent to be recontacted for the donation of physical activity data collected through their privately owned physical activity tracker. Of those, 240 participants eventually shared their physical activity data via Thryve.

#### Open dataset

Participants who owned an activity tracker (or smart watch) from Fitbit or Garmin, and who agreed to share physical activity-related data collected from these devices between January 2019 and December 2020, were eligible for inclusion. Data were collected from 113 participants who shared their physical activity data using privately owned activity trackers (or smart watches) from Garmin and Fitbit.

### Measures

In both SMART 2.0 and 4HAIE, daily steps were collected with a Fitbit Charge 3 or 4 (Fitbit Inc., San Francisco, CA, USA). Participants were asked to wear the wrist-worn device continuously during the trial according to the manufacturer’s recommendations (i.e., to wear the device on one’s non-dominant arm and up to three finger widths above the wrist bone). In the other two samples, data were retrospectively collected using crowdsourcing, i.e., participants consented to share their data collected via wearable devices with the research teams. Specifically, data collection extraction was supported by Thryve in ref. ^24^ and mSpider tool in ref. ^23^. In ref. ^23^, 35% of the participants wore a Fitbit device while the remaining 65% were Garmin users. Of the 240 COVICAT participants who provided step count data measured through physical activity trackers^24^, 70 (29%) used a Fitbit, 134 (56%) owned a Garmin watch, 35 (15%) provided step count via Google Fit, and 5 (2%) through a Nokia device.

#### Rate of change in the physical activity stressor-response process

Following a recent tutorial on physical activity resilience^7^, the rate of change in step count after the stressor was operationalized and implemented as follows. First, we defined the normal range in daily step count as the median number of daily steps during the five and a half months preceding the lockdown. Second, to model the physical activity trajectory following the stressor, we applied concurrent growth models to the step count time series following the beginning of the lockdown. Specifically, we fit the same versions of Generalized Additive Models (GAMs) through the R package *mgcv*^31^ and selected the best-fitting growth model based on the Root Mean Squared Error (RMSE), which tells how close the model’s fitted values were to the observed data points. GAMs are particularly suited for defining the growth model, as they allow for flexible modeling of various non-linear patterns characterizing the time series’ trajectory. In this analysis, the model’s flexibility was specified by setting the number of basis functions (the *k* parameter in the *gam* function) to 20. More practically, this setting allowed each time series to be modeled with up to 20 degrees of freedom, providing sufficient flexibility to capture fluctuations on the order of roughly every 10 days across the observation period (from lockdown enforcement to the end of September). We initially tested *k* = 10 (approximately one fluctuation every 20 days). This yielded results highly comparable to those reported above in the Results section, with a very similar cross-correlation value between local DC and rate of change (–0.23 vs. –0.22), but with the peak correlation shifted slightly (lag –15 vs. lag –11). We ultimately selected *k* = 20, as this specification provided greater sensitivity to shorter-term fluctuations in step count trajectories and resulted in a more proximal alignment between local DC and subsequent changes in step count.

We extended this recently developed method^7^ by quantifying the rate of change in the stressor-response trajectory using the first-order derivative ($\frac{{\rm{d}}y}{{\rm{d}}t}$) of the growth function. The first-order derivative provides insight into the instantaneous rate of change of the step count trajectory, defining the resilience process. Positive values ($\frac{{d}{y}}{{d}{t}}$ > 0) indicate that the function is increasing at that point, suggesting improvement or recovery. Negative values ($\frac{{d}{y}}{{d}{t}}$ < 0) indicate that the function is decreasing, suggesting a decline or setback. A derivative of zero ($\frac{{d}{y}}{{d}{t}}$ = 0) indicates a stationary point where there is no instantaneous change, possibly signifying a moment of equilibrium. The analysis of these derivatives allows a precise understanding of the direction and momentary pace of the stressor-response process over time. The derivatives were extracted with the *gratia* R package^32^.

#### Local Dynamic Complexity

Dynamic complexity (DC) was assessed by analyzing fluctuations in the step time series. The DC score is calculated as the product of two measures: a fluctuation measure, *F*, and a distribution parameter, *D*^18,33^. The fluctuation measure is sensitive to both the amplitude and frequency of changes within the time series, peaking when data dynamics vary greatly from one observation to the next, with large and regular fluctuations (such as between the minimum and maximum values of the outcome). The distribution parameter quantifies the deviation of data points from the range of possible values in the time series, increasing with the occurrence of irregular changes from one observation to the next^18^. We refer to ‘local’ DC because the dynamic complexity scores were computed locally using a 14-day moving window throughout the time series. This means that the DC value on day *n* was derived from the period spanning from day *n*-14 to day *n*. The 14-day window was chosen iteratively: although we initially tested a 7-day window as done in^13^, cross-correlation (see next section on Data Analysis) with the rate of change was stronger using 14 days, yielding greater predictive potential.

#### Pre-lockdown step count (baseline)

Baseline step count was defined as the median number of daily steps recorded between October 1, 2019, and the date of the country-specific lockdown enforcement. Details on the validity criteria for the baseline period (i.e., minimum of three valid weeks) are provided in the Data Analysis section.

### Data analysis

Starting from the initial sample as described in the ‘Participants’ section, we applied the following pre-processing steps:

1. *Identification of valid daily step count data.* When wear time information was available (Smart 2.0 study, 4HAIE study, and Fitbit users from the open dataset), valid days were defined as days with at least 10 hours of wear time^34^ and at least one step count registered. When wear time information was unavailable (COVICAT study and Garmin users from the open dataset), valid days were defined as days with at least one step count registered. In the latter case, we decided not to apply a higher step count threshold (e.g., 150 daily steps as done in^23^) because, in cases where both daily step counts and wear time data were available (e.g., Smart 2.0 and 4HAIE studies), we observed instances where wear time exceeded 10 hours, yet daily steps fell within the range of 1–150. Non-valid days were recoded as missing values.
2. *Recoding upper bound outliers.* Daily step count observations above 40,000 have been recoded to 40,000 steps, as this approach prevents extreme values from influencing the results while preserving the integrity of the data by retaining realistic upper limits for step count.

Participants were drawn from the parent studies and included in the current analysis if they satisfied the following criteria:

1. *Valid baseline period:* Participants had at least three valid weeks of data during the pre-pandemic baseline period (October 1, 2019, to the date of the country-specific lockdown enforcement). A valid week was defined, based on prior research^35^, as including at least one weekend day and two weekdays. The three-week requirement was chosen as a compromise to ensure a sufficiently stable baseline characterization while maximizing the number of participants included. This conservative threshold exceeds the minimum number of days typically recommended to obtain reliable step count estimates with accelerometry (i.e., 4-6 days)^36,37^.
2. *Valid home confinement period:* Participants had valid data for all weeks within the four-week period following the country-specific lockdown enforcement, during stricter home confinement. For this period, a valid week was defined as including at least 3 valid days. Weekdays and weekend days were not differentiated, as the home confinement removed typical distinctions between them; therefore, all days within a week were treated equally.
3. *Valid recovery period:* Participants had at least 70% of valid weeks during the recovery period (four weeks after the country-specific lockdown enforcement through September 30, 2020). A valid week was defined as including at least one weekend day and two weekdays, consistent with prior research^35^.
4. *Missing data threshold:* To ensure sufficient data for reliable imputation and modeling, only participants with less than 20% missing values in their post-lockdown time series (from the date of the country-specific lockdown enforcement to September 30, 2020) were included.
5. *Step count perturbation:* After modeling growth functions (as described in the measures section), participants were included only if they experienced a perturbation of at least a 10% decrease in step counts during the 30 days following the lockdown relative to their median pre-lockdown step count level. This additional inclusion criterion is based on the study’s research question, which necessitates a perturbation in the system following the occurrence of the stressor.

Descriptive statistics of missing values, visualizations of their distribution, and trends in step counts during the post-stressor phase for the final analytical sample are presented in the supplemental materials (Supplementary Table 7, Supplementary Figs. 2–5).

The rate of change in the physical activity stressor-response process (dependent variable) was calculated as described in the measures section. Missing value imputation was not applied directly prior to modeling, as the growth model’s predicted values inherently provided estimates for originally missing observations. The threshold for defining a return to pre-stressor levels was operationalized as the median pre-lockdown step count, multiplied by a factor of 1.10 to account for the typically higher physical activity volumes in spring and summer compared to fall and winter^38,39^.

Local dynamic complexity (independent variable) was calculated with an algorithm specifically designed to detect critical fluctuations in time series, implemented through the R package *casnet*^40^. The function used to calculate the local DC metric does not permit missing values in the input time series. This is an important constraint, as missing values could result in an invalid metric, particularly for the distribution parameter, due to unequally spaced observations. To address this, missing daily step values were imputed using the Kalman Filter method, which is recommended for univariate time series imputation^41^. After data exploration and visual inspection of the cross-correlation plot for local DC calculated using 7-day and 14-day moving windows, we selected the 14-day window as it provided a more interpretable solution.

Before testing our main hypotheses, we examined the cross-correlation between local DC and the rate of change in the step count trajectory following the stressor. Cross-correlation is a statistical measure that determines the relationship between two time series, accounting for potential time lags. It is useful for identifying time delays and understanding how one series influences another. In our case, it aimed to identify the time lag at which local DC had its largest effect on the rate of change.

After the preliminary examination of the cross-correlations, we tested our hypotheses using a multilevel approach, implemented through the R package *lme4*^42^. The model included fixed effects for time (i.e., the progressive number of days since the start of the lockdown), local DC, and the quadratic term of local DC. Individual differences in the rate of change in the step count trajectory were accounted for by including a random intercept for the participant identifier. Adopting a “maximal approach”^43^, random slopes were also estimated for time, local DC, and its quadratic term. The selection of these predictors, as both fixed and random effects, was pre-specified before running the analyses and guided by prior work^13^. Although the quadratic term for local DC was not included in that earlier study, we added it here because the findings suggested that the association between local DC and sudden changes in physical activity may follow a non-linear pattern: specifically, local DC was positively related to sudden losses but showed a weaker negative, non-significant associations with sudden gains. To assess this extension, we compared our final model with an otherwise identical model that excluded the quadratic term (both as a fixed and random effect). Results based on the Bayesian Information Criterion (BIC) indicated a better fit for the model, including the quadratic term (BIC decreased from 467,878 to 466,562). We focused on BIC as our performance metric because it favors more parsimonious models compared to other criteria, such as the Akaike Information Criterion (AIC)^44^. The linear and quadratic terms for local DC were taken from the point in time associated with the largest cross-correlation value. Finally, the pre-lockdown median number of steps (i.e., baseline steps) was included as a between-person variable to account for individual differences in physical activity level, under the assumption that more active participants would recover faster. The linear and quadratic terms for local DC were standardized, while time was scaled but not centered.

The multilevel equation, in the *lme4* syntax, is specified as follows:

$${Rate\; of\; change} \sim {PreLockdown\; steps}+{Time}+{LDC}+{({LDC})}^{2}+({Time}+{LDC}+{({LDC})}^{2}{|Participant\; ID})$$

For the sensitivity analysis, we ran four additional models—one for each data source (Smart 2.0, 4HAIE, COVICAT, and the Open dataset)—to test whether the effect of local DC on the rate of change was moderated by the study data source (operationalized as a dummy variable). We also conducted a sensitivity analysis that included only complete and partially complete cases. In addition, we examined whether device brand moderated the effect of local DC on the rate of change. Finally, we tested a model including age as a predictor. For this latter analysis, participants from the Open dataset were excluded, as person-specific age information was not available. The results of these sensitivity analyses are presented in the Supplementary Tables 2–6.

## Supplementary information

Supplementary Information

## Data Availability

The data used in the analysis are available at https://osf.io/gsmhk/.

## Code availability

The R scripts used for the analysis are available at https://osf.io/gsmhk/.
