# Supplementary material for: Predicting recovery after stressors using step count data derived from activity monitors
Source: NPJ Digit Med. 2025 Oct 9;8:606. doi: 10.1038/s41746-025-01998-0 (PMC12511605; doi:10.1038/s41746-025-01998-0)
Supplement: Supplementary file 1 — Supplementary Information [file 41746_2025_1998_MOESM1_ESM.pdf]

Supplementary materials to the manuscript:

Predicting Recovery After Stressors Using Step Count Data Derived From Activity Monitors

Authors:

Dario Baretta, Sarah Koch, Joren Buekers, Judith Garcia-Aymerich, Lenka Knapova, Steriani Elavsky, Job Godino, Merlijn Olthof, Anna Lichtwarck-Aschoff, Ruud den Hartigh, & Guillaume Chevance

Supplementary Table 1. Descriptive statistics of key variables by data source.

	Smart 2.0 study (California)	4HAIE study (Czech Republic)	COVICAT study (Spain)	Open Dataset (Norway)	Overall
**Pre-lockdown steps (baseline)**	9,475 [7,425, 10,992]; 9,413	12,071 [9,321, 14,710]; 12,637	10,492 [8,168, 12,047]; 10,438	9,550 [6,927, 11,201]; 9,329	10,167 [8,063, 12,188]; 10,544
**Rate of change**	5 [-1, 14]; 6	7 [-2, 25]; 11	12 [3, 31]; 16	18 [2, 24]; 16	8 [-1, 23]; 11
**Post-lockdown steps**	5,889 [4,083, 7,181]; 6,165	11,196 [8,921, 14,573]; 11,903	8,278 [6,316, 9,915]; 8,157	8,604 [6,728, 10,697]; 8,825	8,267 [5,939, 10,883]; 8,662
**Dynamic complexity**	0.044 [0.033, 0.060]; 0.049	0.045 [0.033, 0.068]; 0.052	0.048 [0.032, 0.060]; 0.050	0.063 [0.048, 0.085]; 0.070	0.048 [0.034, 0.068]; 0.053

Note. Data shown as "Median [Q1, Q3]; Mean". Dynamic complexity is unstandardized.

Supplementary Figure 1. Spaghetti plot illustrating random intercepts and slopes for the non-linear association between local dynamic complexity and the rate of change.

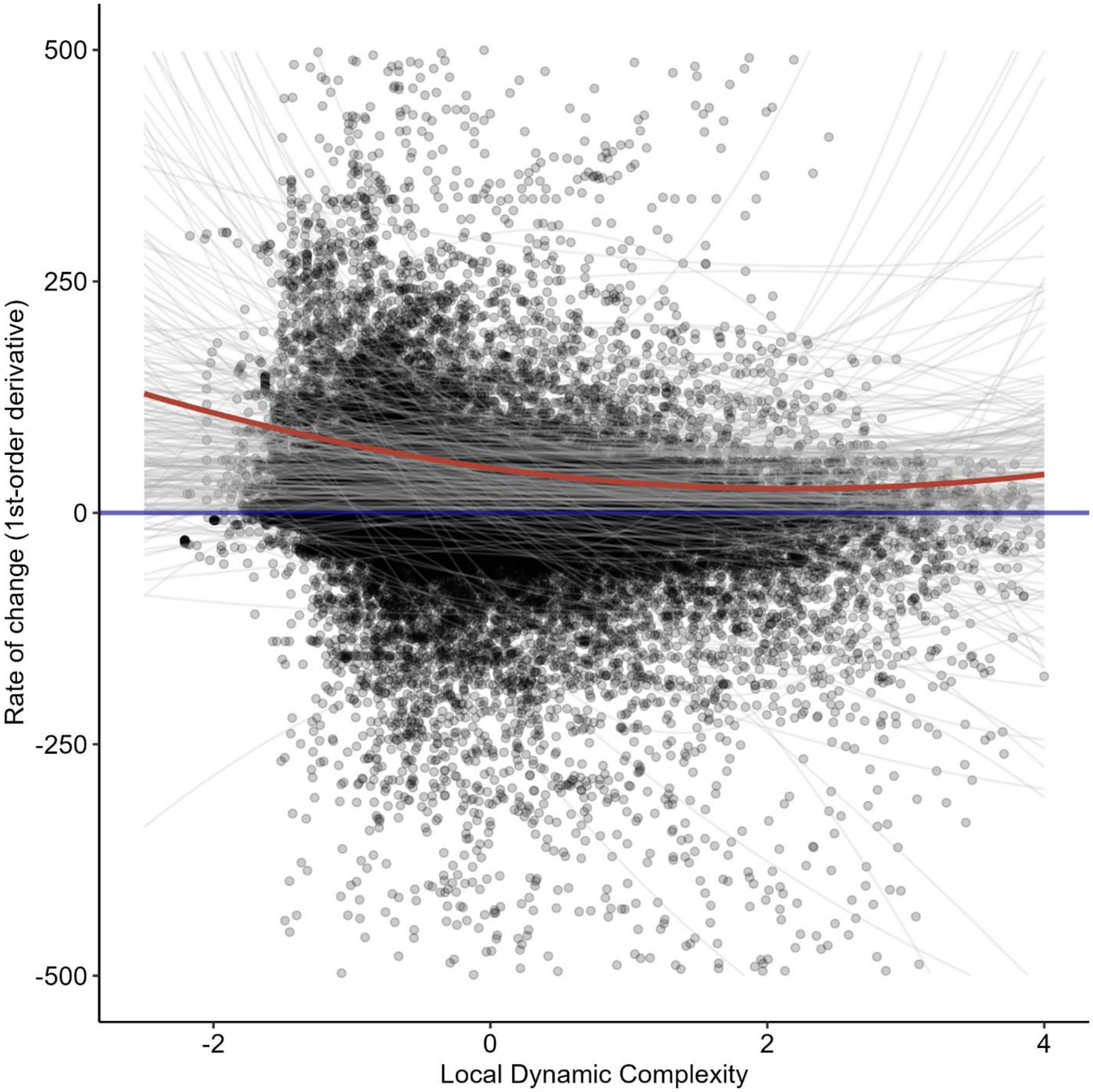

Note. The red bold line represents the fixed effect (intercept and combined linear + quadratic terms) from the model. Local dynamic complexity values on the x-axis are standardized.

Supplementary Table 2. Sensitivity analysis: summary of multilevel models controlling for the moderating effect of each data source location (country) on the association between local dynamic complexity and rate of change.

**DV is rate of change**	**Main model**	**Smart 2.0 study (California)**	**4HAIE study (Czech Republic)**	**COVICAT study (Spain)**	**Open Dataset (Norway)**
Fixed effects - **Est. (SE), 95% CI**					
**Intercept**	**48.45 (4.89)**	**50.74 (4.93)**	**49.08 (4.98)**	**46.69 (4.91)**	**47.98 (4.93)**
	**[38.85, 58.04]**	**[41.08, 60.40]**	**[39.33, 58.84]**	**[37.07, 56.32]**	**[38.32, 57.64]**
**Pre-lockdown median steps**	-0.81 (1.19)	-1.52 (1.21)	-0.51 (1.29)	-0.71 (1.17)	-0.66 (1.21)
	[-3.15, 1.52]	[-3.88, 0.84]	[-3.03, 2.02]	[-3.00, 1.58]	[-3.02, 1.71]
**Time**	**-43.51 (5.35)**	**-43.48 (5.34)**	**-43.52 (5.35)**	**-43.50 (5.35)**	**-43.51 (5.35)**
	**[-53.98, -33.03]**	**[-53.96, -33.01]**	**[-54.00, -33.04]**	**[-53.98, -33.02]**	**[-53.99, -33.03]**
**Local DC**	**-20.42 (2.73)**	**-24.01 (3.04)**	**-19.20 (3.00)**	**-18.55 (2.91)**	**-20.50 (2.87)**
	**[-25.78, -15.07]**	**[-29.97, -18.05]**	**[-25.09, -13.32]**	**[-24.25, -12.85]**	**[-26.12, -14.88]**
**Local DC^2**	**4.67 (1.14)**	**4.68 (1.14)**	**4.67 (1.14)**	**4.66 (1.14)**	**4.67 (1.14)**
	**[2.44, 6.90]**	**[2.45, 6.91]**	**[2.44, 6.90]**	**[2.43, 6.89]**	**[2.44, 6.90]**
**Country**	-	**-6.84 (2.56)**	-2.17 (2.85)	**8.28 (2.88)**	2.93 (3.30)
	-	**[-11.86, -1.83]**	[-7.75, 3.41]	**[2.64, 13.92]**	[-3.54, 9.40]
**Local DC*Country**	-	**10.48 (4.09)**	-4.24 (4.33)	-8.72 (4.77)	0.46 (5.38)
	-	**[2.47, 18.50]**	[-12.73, 4.25]	[-18.07, 0.64]	[-10.10, 11.01]

Random effects					
**SD (Intercept)**	70.89	70.23	71.07	70.58	70.97
**SD (Time)**	78.42	78.38	78.41	78.44	78.42
**SD (Local DC)**	39.85	39.39	39.86	39.78	39.92
**SD (Local DC^2)**	16.11	16.10	16.11	16.12	16.11
Model metrics					
**Num.Obs.**	39,401	39,401	39,401	39,401	39,401
**R2 Marg.**	0.067	0.071	0.068	0.071	0.067
**R2 Cond.**	0.325	0.326	0.325	0.327	0.326
**BIC**	466,547.1	466,545.5	466,558.2	466,546.7	466,558.0
**ICC**	0.3	0.3	0.3	0.3	0.3

***Note*.** The *Main model* refers to the model presented in the manuscript. Bold values indicate significance based on 95% confidence intervals. *R^2 Marginal* represents the variance in the outcome explained by fixed effects, while *R^2 Conditional* represents the variance explained by the full model. * denotes interaction terms.

Supplementary Table 3. Sensitivity analysis: summary of multilevel models controlling for data source location (country), modeled as a random effect.

DV is rate of change		Main model	+ Site-level random intercept	+ Site-level random intercept and slope (Local DC)
Fixed effects - **Est. (SE), 95% CI**				
**Intercept**		**48.45 (4.89)**	**49.22 (5.37)**	**49.14 (5.32)**
		**[38.85, 58.04]**	**[38.69, 59.75]**	**[38.71, 59.56]**
**Pre-lockdown median steps**		-0.81 (1.19)	-0.92 (1.24)	-0.94 (1.24)
		[-3.15, 1.52]	[-3.36, 1.52]	[-3.37, 1.50]
**Time**		**-43.51 (5.35)**	**-43.54 (5.35)**	**-43.50 (5.35)**
		**[-53.98, -33.03]**	**[-54.02, -33.06]**	**[-53.98, -33.03]**
**Local DC**		**-20.42 (2.73)**	**-20.41 (2.73)**	**-20.94 (3.72)**
		**[-25.78, -15.07]**	**[-25.77, -15.06]**	**[-28.23, -13.64]**
**Local DC^2**		**4.67 (1.14)**	**4.67 (1.14)**	**4.67 (1.14)**
		**[2.44, 6.90]**	**[2.44, 6.90]**	**[2.44, 6.90]**
Random effects				
**ID**	**SD (Intercept)**	70.89	70.39	70.44
**ID**	**SD (Time)**	78.42	78.42	78.41
**ID**	**SD (Local DC)**	39.85	39.85	39.55
**ID**	**SD (Local DC^2)**	16.11	16.11	16.11
**Site**	**SD (Intercept)**	-	4.52	4.25
**Site**	**SD (Local DC)**	-	-	5.02
Model metrics				
**Num.Obs.**		39401	39401	39401
**R2 Marg.**		0.067	0.067	0.069
**R2 Cond.**		0.325	0.326	0.326

**BIC**	466,547.1	466,551.4	466,559.8
**AIC**	466,409.8	466,405.5	466,405.3
**ICC**	0.3	0.3	0.3

Note. The Main model refers to the specification presented in the manuscript.

- \+ *Site-level random intercept*: adds a random intercept for study site to the participant-level random intercepts and slopes (R specification: Rate of change ~ Pre-lockdown steps + Time + LDC + (LDC)^2 + (Time + LDC + (LDC)^2 | Participant ID) + (1 | site)).
- \+ *Site-level random intercept and slope (LDC)*: further adds a site-level random slope for dynamic complexity (R specification: Rate of change ~ Pre-lockdown steps + Time + LDC + (LDC)^2 + (Time + LDC + (LDC)^2 | Participant ID) + (LDC | site)).

Bold values indicate significance based on 95% confidence intervals. *R^2 Marginal* represents the variance in the outcome explained by fixed effects, while *R^2 Conditional* represents the variance explained by the full model.

Supplementary Table 4. Sensitivity analysis: comparison of the main model presented in the manuscript with alternative models using datasets limited to complete or partially complete cases.

DV is rate of change	Main model	Complete cases model	Partially complete cases model
Fixed effects - Est. (SE), 95% CI			
Intercept	**48.45 (4.89)**	**53.00 (5.32)**	**50.78 (5.07)**
	**[38.85, 58.04]**	**[42.56, 63.43]**	**[40.84, 60.72]**
Pre-lockdown median steps	-0.81 (1.19)	-1.19 (1.52)	-1.29 (1.27)
	[-3.15, 1.52]	[-4.17, 1.78]	[-3.77, 1.20]
Time	**-43.51 (5.35)**	**-48.65 (5.85)**	**-46.09 (5.59)**
	**[-53.98, -33.03]**	**[-60.12, -37.17]**	**[-57.05, -35.13]**
Local DC	**-20.42 (2.73)**	**-16.52 (2.53)**	**-18.16 (2.67)**
	**[-25.78, -15.07]**	**[-21.49, -11.56]**	**[-23.40, -12.92]**
Local DC^2	**4.67 (1.14)**	**3.49 (1.15)**	**3.89 (1.07)**
	**[2.44, 6.90]**	**[1.23, 5.75]**	**[1.80, 5.99]**
Random effects			
SD (Intercept)	70.89	76.21	73.49
SD (Time)	78.42	84.87	82.03
SD (Local DC)	39.85	35.85	38.78
SD (Local DC^2)	16.11	15.63	14.90
Model metrics			
Num.Obs.	39,401	29,889	36,662
R2 Marg.	0.067	0.068	0.067
R2 Cond.	0.325	0.342	0.329
BIC	466,547.1	353,421.5	433,848.4
ICC	0.3	0.3	0.3

Note. The *Main model* refers to the model presented in the manuscript. The *Complete cases model* excludes entries where (1) raw step count observations were missing, or (2) local dynamic complexity values were derived from a 14-day rolling window with any missing values. The *Partially complete cases model* excludes entries where (1) raw step count observations were missing, or (2) local dynamic complexity values were derived from a 14-day rolling window with more than five missing values. Bold values indicate significance based on 95% confidence intervals. *R^2 Marginal* represents the variance in the outcome explained by fixed effects, while *R^2 Conditional* represents the variance explained by the full model.

Supplementary Table 5. Multilevel model summary including the moderating effect of type of activity monitor (Fitbit) on the association between local dynamic complexity and rate of change.

	**Fixed effects**		**Random effects**
**DV is rate of change**	**Est. (SE)**	**95% CI**	**SD**
**Intercept**	**50.69 (5.64)**	**[39.65, 61.74]**	70.92
**Time**	**-43.51 (5.35)**	**[-53.99, -33.03]**	78.43
**Local DC**	**-24.09 (5.29)**	**[-34.45, -13.73]**	39.88
**Local DC^2**	**4.66 (1.14)**	**[2.43, 6.90]**	16.11
**Pre-lockdown median steps**	-0.64 (1.21)	[-3.01, 1.73]	-
**Fitbit**	-2.66 (3.31)	[-9.15, 3.83]	-
**Local DC*Fitbit**	4.36 (5.38)	[-6.19, 14.91]	-
Number observations = 39,401; ICC = 0.4; R2 Marg. = 0.067; R2 Cond. = 0.395			

Note. Bold values indicate significance based on 95% confidence intervals. R^2 *Marginal* represents the variance in the outcome explained by fixed effects, while R^2 *Conditional* represents the variance explained by the full model.

Supplementary Table 6. Multilevel model summary including age as a covariate.

DV is rate of change	Main model	Model with age
Fixed effects - Est. (SE), 95% CI		
Intercept	**50.85 (5.61)**	**50.88 (5.58)**
	**[39.85, 61.85]**	**[39.95, 61.81]**
Pre-lockdown median steps	0.07 (1.06)	-0.33 (1.05)
	[-2.01, 2.15]	[-2.40, 1.73]
Time	**-46.16 (5.97)**	**-46.17 (5.96)**
	**[-57.86, -34.47]**	**[-57.86, -34.48]**
Local DC	**-19.16 (2.95)**	**-19.16 (2.95)**
	**[-24.94, -13.39]**	**[-24.94, -13.39]**
Local DC^2	**3.97 (1.18)**	**3.97 (1.18)**
	**[1.67, 6.28]**	**[1.67, 6.28]**
Age	-	**3.18 (1.07)**
	-	**[1.07, 5.28]**
Random effects		
SD (Intercept)	75.33	74.85
SD (Time)	80.73	80.72
SD (Local DC)	39.65	39.64
SD (Local DC^2)	15.40	15.40
Model metrics		
Num.Obs.	33,137	33,137
R2 Marg.	0.083	0.084
R2 Cond.	0.380	0.380
BIC	383,891.8	383,891.9
ICC	0.3	0.3

Note. The main model refers to the model presented in the manuscript but with the exclusion of the Norway data source, as age could not be linked to physical activity data in this sample. Bold values indicate significance based on 95% confidence intervals. *R^2 Marginal* represents the variance in the outcome explained by fixed effects, while *R^2 Conditional* represents the variance explained by the full model.

Supplementary Table 7. Summary of missing value descriptive statistics.

Missing values	Overall<br>N = 226	Smart 2.0<br>N = 77	4HAIE<br>N = 65	COVICAT<br>N = 48	Open dataset<br>N = 36
**Median (IQR)**	7 (1, 16)	8 (2, 18)	10 (5, 25)	4 (0, 13)	0 (0, 7)
**Mean (SD)**	10 (11)	11 (10)	14 (13)	8 (9)	5 (8)

Note. Median, interquartile range (IQR), mean, and standard deviation (SD) of missing values per time series, reported for the entire dataset and stratified by data source.

Supplementary Figure 2. Distribution of the step count missing values in the timeseries included in the main analysis (Smart 2.0 and 4HAIE studies).

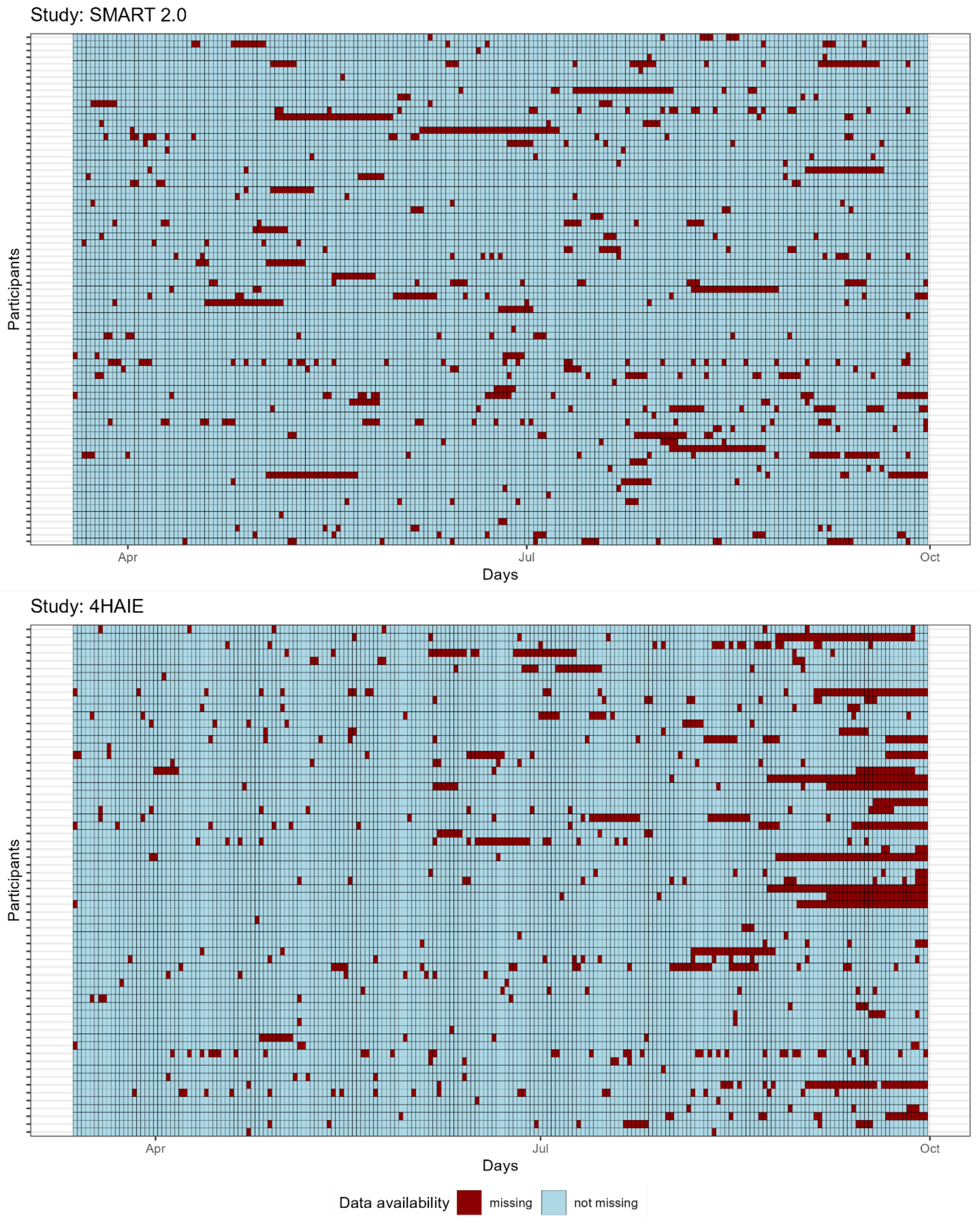

Supplementary Figure 3. Distribution of the step count missing values in the timeseries included in the main analysis (COVICAT study and open dataset).

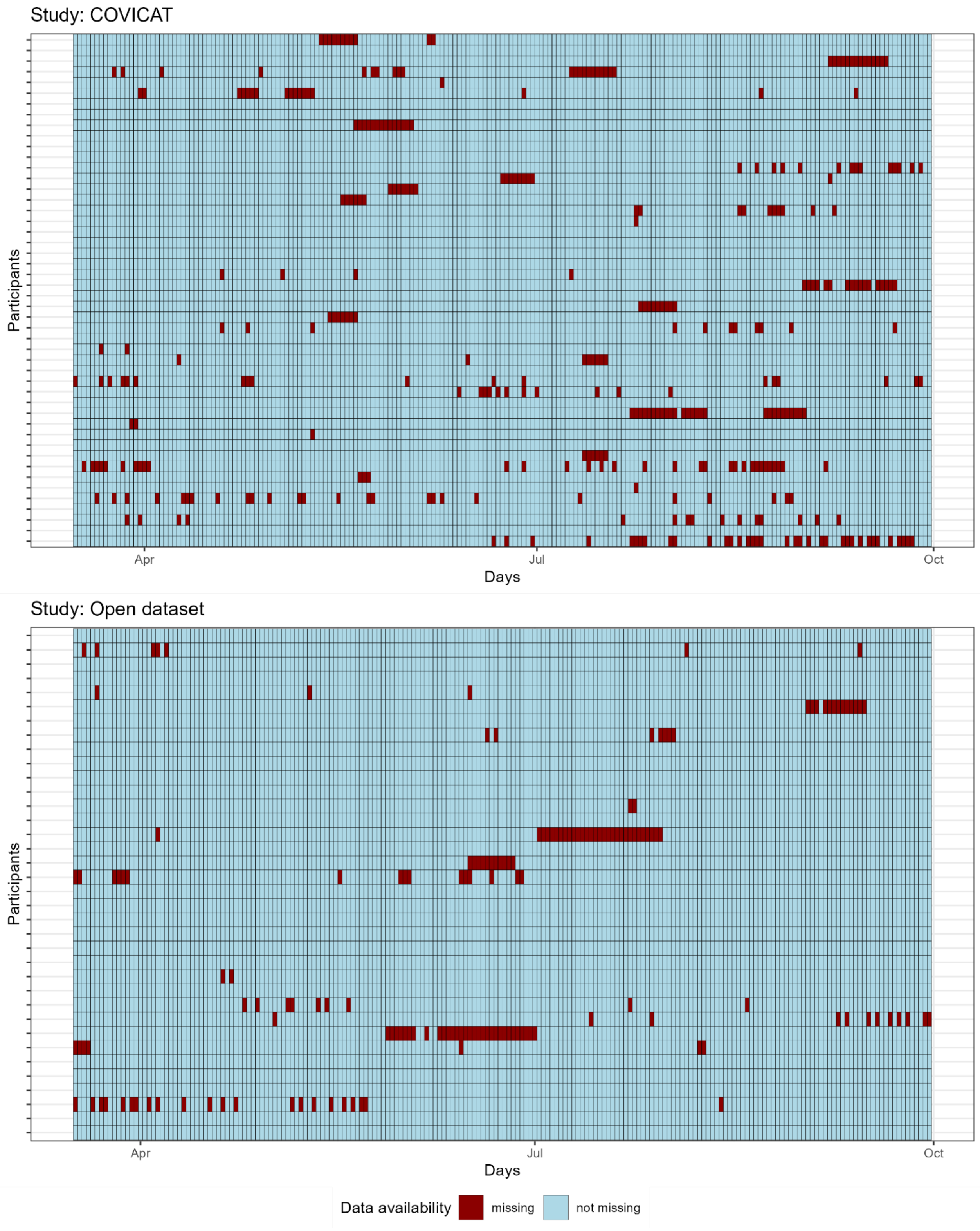

Supplementary Figure 4. Changes in step count over time following the first COVID-19 Lockdown (Smart 2.0 and 4HAIE studies)

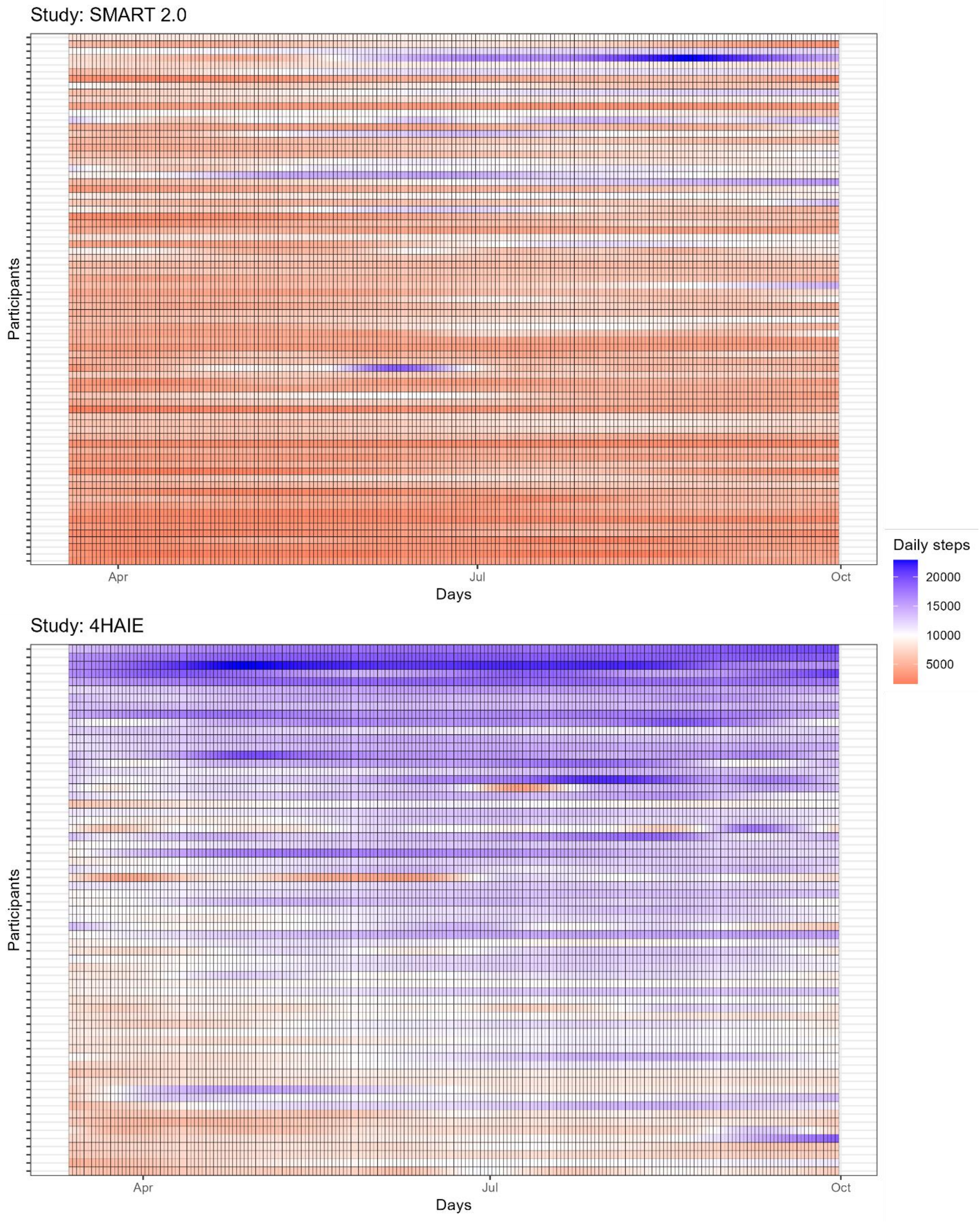

Note. To highlight seasonal trends and minimize day-to-day variations, the colors in the heatmaps represent the predicted daily step count from the person-specific growth models.

Supplementary Figure 5. Changes in step count over time following the first COVID-19 Lockdown (COVICAT study and open dataset)

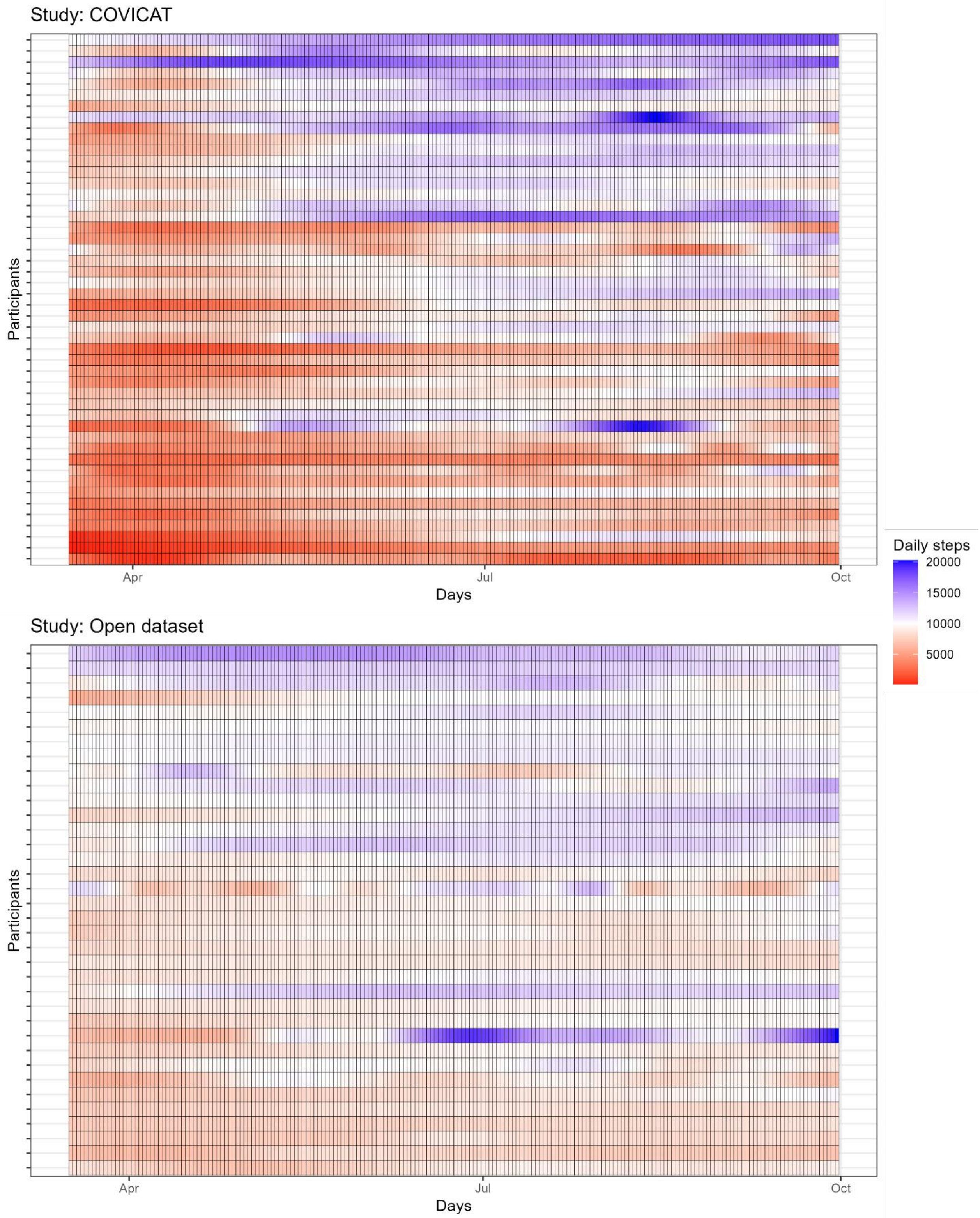

Note. To highlight seasonal trends and minimize day-to-day variations, the colors in the heatmaps represent the predicted daily step count from the person-specific growth models.
